# Influence of Plasma Treatment Parameters on the Structural-Phase Composition, Hardness, Moisture-Resistance, and Raman-Enhancement Properties of Nitrogen-Containing Titanium Dioxide

**DOI:** 10.3390/ma15238514

**Published:** 2022-11-29

**Authors:** Arsen E. Muslimov, Makhach Kh. Gadzhiev, Vladimir M. Kanevsky

**Affiliations:** 1 Federal Scientific Research Centre “Crystallography and Photonics” of Russian Academy of Sciences, Shubnikov Institute of Crystallography, Moscow 119333, Russia; 2 Joint Institute for High Temperatures, Russian Academy of Sciences, Moscow 125412, Russia

**Keywords:** titanium, sapphire, magnetron deposition, low-temperature plasma, hydrophobicity, hardness, rutile, Raman enhancement, nitrogen

## Abstract

The paper shows, for the first time, the prospects of treatment with a quasi-equilibrium low-temperature nitrogen plasma in an open atmosphere for the formation of super-hard, super-hydrophobic TiN/TiO_2_ composite coatings with pronounced Raman-enhancement properties. X-ray diffractometry (XRD), scanning electron microscopy (SEM), atomic force microscopy (AFM), and Raman spectroscopy, as well as the analysis of hardness and moisture-resistance properties, are used as analytical research methods. During plasma treatment of titanium films on sapphire with a mass average temperature of 4–6 kK, an X-ray amorphous hydrophilic titanium oxide film with a low nitrogen content is formed. The nitrogen content in titanium oxide films increases with increasing treatment temperature up to 6–7 kK. In this case, an X-ray amorphous hydrophobic film is formed. With a further increase in temperature to 7–10 kK, a TiN/TiO_2_ composite structure based on polycrystalline rutile is formed with increased hydrophobicity and pronounced Raman enhancement properties due to the effective excitation of surface plasmon polaritons. The presence of the crystalline phase increases the dephasing time, which determines the quality of the resonance and the achievable amplification of the electromagnetic field near the TiN inclusions. All treated films on sapphire have a super-hardness above 25 GPa (Vickers hardness test) due to high grain size, the presence of nitrogen-containing inclusions concentrated along grain boundaries, and compressive stresses.

## 1. Introduction

Plasma treatment, as one of the methods of surface modification, is the most used today [1,2,3,4,5,6]. The advantage is the ability to process objects of complex shapes. In addition, economy and environmental friendliness are important parameters. High-temperature (temperature up to 10^8^ K) plasma is characterized by a high level of ionization. The use of high temperatures are hampered by the low level of control of the degree of impact and the rapid destruction of materials. For this reason, low-temperature plasma is widely used. The low-temperature plasma can be cold and thermal. A distinctive feature of cold plasma [7] is the low temperature (25–100 °C) of heavy particles and the high temperature of electrons (up to 10^5^ °C). Cold plasma with a low temperature close to physiological (30–40 °C) is used in biomedicine [7]. Cold plasma with an elevated temperature is used to modify the surface of materials. It is used, for example, to activate the surface of catalysts, to control the topography and physical and chemical properties of the surface of materials [8]. Low-temperature plasma nitriding at a temperature of 450 °C is used to improve the hardness and wear resistance of steel [4]. It seems to us that devices generating low-temperature thermal (up to 10^4^ K) plasma have a high applied potential, since they can be used as a universal heat carrier and reagent at the same time. The relatively high temperature and activity of plasma-forming gases make it possible to modify the surface of metal products, including refractory materials, in a short period of time. One of the most common and affordable materials widely used in modern technologies is titanium, as well as compounds based on it. The most important relevant indicators of any prospective material are its hardness, corrosion, and moisture resistance, as well as high oxygen resistance, especially when operating at high temperatures and in aggressive environments. Such high-performance materials include titanium oxides and oxynitrides. Among the main phases of titanium dioxide, rutile has the highest microhardness (about 14 GPa) [9,10,11]. This is not the limit, and it has been shown in [12] that at a pressure of 60–70 GPa and a temperature of 1100 K, it is possible to obtain a titanium dioxide phase with a cotunite structure and a microhardness of 38 GPa. The authors explain the result obtained by the influence of compressive residual stresses. In addition [13], a method of strengthening titanium alloys by nitriding is known. It seems to us that the treatment of a titanium surface with a low-temperature nitrogen plasma in an open atmosphere can be the most effective way to form titanium oxynitrides with increased hardness. If we talk about moisture resistance, the high sorption of OH groups by the surface of titanium dioxide [14], on the contrary, contributes to its hydrophilicity. However, it is known from the literature that hydrophilic properties, together with interfacial chemical interaction, can be radically affected by roughness, surface micromorphology [15], crystal structure, impurity composition, and monophasicity [16]. It is shown in [16] that on titanium dioxide surfaces with similar roughness values, depending on the phase composition, completely opposite states can be realized: single-phase surface is characterized by hydrophobicity, and in the case of polymorphic structure it changes to a hydrophilic state. The effect of nitrogen doping on the moisture-resistant properties of the TiO_2_ uniaxial nanostructures array was investigated in [17]. Doping with nitrogen affected only the parameters of TiO_2_ nanorods and, in turn, contributed to hydrophobicity by reducing the area of contact with water. For continuous titanium dioxide films of mixed-phase composition with a low level of doping with nitrogen (less than 0.1%), a hydrophilic state is realized [17]. The data presented in the literature confirm the dependence of the hydrophilic properties of titanium dioxide films on the structural-phase and impurity composition, roughness, and surface morphology. A comprehensive analysis of the relationship between the parameters of plasma (thermal plasma) treatment and the structure and properties of materials has not been found in the literature. Apparently, weak control of the degree of plasma exposure to the material being processed hinders the widespread introduction of the plasma processing technique: the probability of sample destruction is high. However, for such refractory materials as titanium, it can be highly effective. One should also keep in mind the possibility of the formation of a TiO_2_ dielectric–TiN semiconductor composite structure during the action of nitrogen plasma in an open atmosphere on the titanium surface. Composite and heterostructures based on TiO_2_ are used as surface-enhanced Raman spectroscopy (SERS) substrates [18,19]. It should be noted that the influence of the crystal structure of TiO_2_ on its Raman-active properties has not been studied. Strengthening is achieved mainly by the deposition of an active metal layer of silver or gold on nanostructures (nanotubes, nanowalls, etc.) of dielectric TiO_2_. An increase in the intensity of Raman scattering of a substance adsorbed on the surface of a metal/dielectric composite is promoted by surface plasmon resonance. Gold or silver is traditionally used as the active metal layer, since they are distinguished by high chemical stability and high efficiency of excitation of surface plasmon polaritons. However, the high cost of such materials and the complexity of synthesizing such hierarchical systems require the search for new approaches and systems. In our opinion, the TiN/TiO_2_ composite synthesized by plasma treatment of titanium may have prospects for application as a SERS substrate. The absorption spectrum of TiN [20] extends from the UV to the near-infrared radiation range, which indicates the metallic nature of the conductivity. This feature makes it possible to observe [21] plasmon effects in titanium nitride nanoparticles in the visible and infrared regions. In particular, there are data on localized surface plasmon resonance directly on TiN nanoparticles in a TiN_x_O_y_ matrix [22].

Accordingly, in the present work, the relationship between the parameters of plasma (thermal nitrogen plasma in an open atmosphere) surface treatment of titanium coatings on their structural-phase composition, hardness, moisture-resistance, and Raman-enhancement properties is investigated.

## 2. Materials and Methods

A 500 nm-thick titanium layer was applied to the sapphire plate. The plate was pre-polished chemo-mechanically. The film was applied by magnetron sputtering. In the next step, the samples were treated in nitrogen plasma. The process was conducted in an open atmosphere. The source of nitrogen plasma was the generator described in detail in [23]. Spectral methods were used to determine plasma flow parameters. Scanning was performed using AvaSpec 2048 spectrometer (Eerbeek, The Netherlands). To measure temperature with the Boltzmann exponent method, there were a sufficient number of NI atomic nitrogen lines in the nitrogen plasma spectra [24]. Samples were obtained at the following mass-average plasma temperatures: type I at 4–6 kK; type II at 6–7 kK; type III at 7–10 kK). To prevent the destruction of the samples, a short-term treatment of 1 min was used. To compare the processing results, we use a commercial titanium plate and a rutile film on sapphire obtained by the high-temperature annealing of titanium in an open atmosphere. The hardness of the samples is studied using a NanoScan-3D scanning nanohardness tester (Optics11 Life, Troitsk, Russia). The microhardness value for the samples is determined by dynamic indentation testing. The Vickers microhardness value is calculated by averaging over indents obtained from the area of 50 µm × 50 µm, with a load of 1 to 50 mN.

For microscopic studies, the JCM-6000 desktop scanning electron microscope (SEM, Tokyo, Japan) are used: the acceleration voltage is 15 keV, high vacuum mode. The electron microscope is equipped with an energy-dispersive X-ray (EDX) microanalyzer: the acceleration voltage is 10 keV.

Topographic studies and roughness calculations were performed using the Ntegra Prima complex. Structural analysis was performed using X’PERTPRO diffractometer (PANalytical, Almelo, The Netherlands). To analyze the moisture resistance properties, the sessile droplet method was used under the following conditions: relative humidity 35–40%, water drop size 5 mm^3^. To achieve a stable drop state, measurements were made 30 s after application. The procedure for measuring the contact angle is described in detail in [25]. For each sample, 5 regions were examined and the results were averaged.

Raman spectra are recorded on an Ntegra Spectra (NT-MDT) facility (Zelenograd, Russia) at a diode laser wavelength of 532 nm, 20 mW power, and a beam spot of ~50 microns in diameter. A 1 mM solution from Methylene Blue (MB) is prepared to measure SERS spectra.

## 3. Results and Discussion

### 3.1. Analysis of the State of Nitrogen Plasma in a Discharge

**Figure 1:**
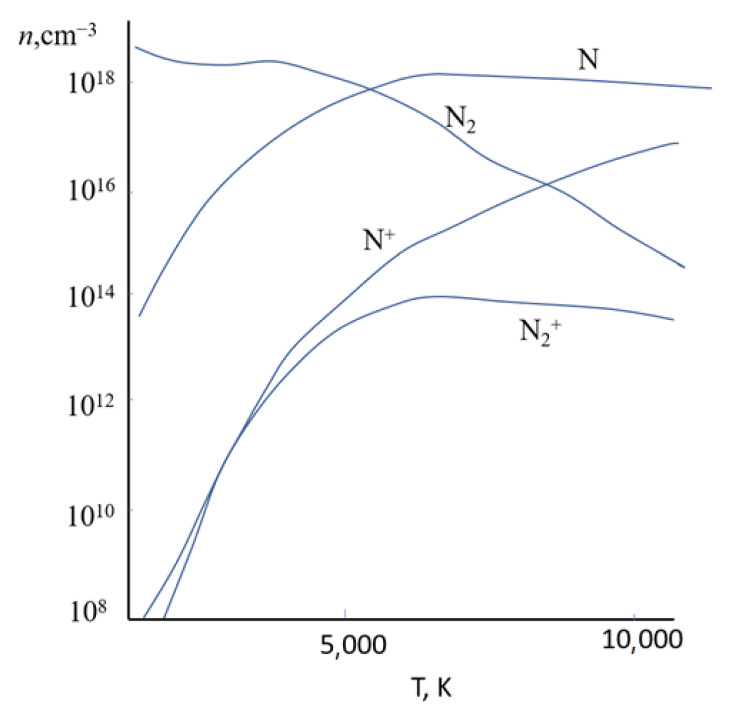
Composition of nitrogen plasma according to the data of [26,27].

In the first stage, electron density and plasma temperature are evaluated. Calculations showed that at the nozzle exit at a current of 250 A, the temperature and electron density in the near-axis nitrogen plasma are T_e_ = 10 kK, n_e_ = 5 × 10^16^ cm^−^^3^. The composition of nitrogen plasma includes: N^2^ molecules in the ground and electronically excited states; N atoms in the ground and electronically excited states; molecular ions N^2+^; atomic ions N^+^; doubly charged nitrogen ions N^2+^. Determining the concentrations of the nitrogen plasma composition components requires solving a complex system of equations; therefore, the results obtained in these studies are different. The average parameters are given in Figure 1 [26,27]. Comparison with the data of [28] demonstrates quantitative differences; however, there are general trends in the temperature dependence of the concentration of individual components of the nitrogen plasma. As expected, the content of the molecular component decreases as the plasma temperature increases. Ionization of nitrogen molecules was detected during the transition from mode I to mode II; however, with a further increase in temperature, the number of molecular ions does not change. The number of neutral nitrogen atoms also increases only initially. On the contrary, the number of ionized nitrogen atoms multiplies, increases, and peaks in mode III.

### 3.2. Studies of the Structural-Phase and Elemental Composition

According to the EDX data, all samples are oxidized titanium coatings with different nitrogen contents. It is difficult to analyze the oxygen content in the films, since the sapphire substrate also contains oxygen and introduces a significant error in the quantitative analysis. For this reason, the N/Ti ratio (at%) is studied according to EDX data (Table 1).

**Figure 2:**
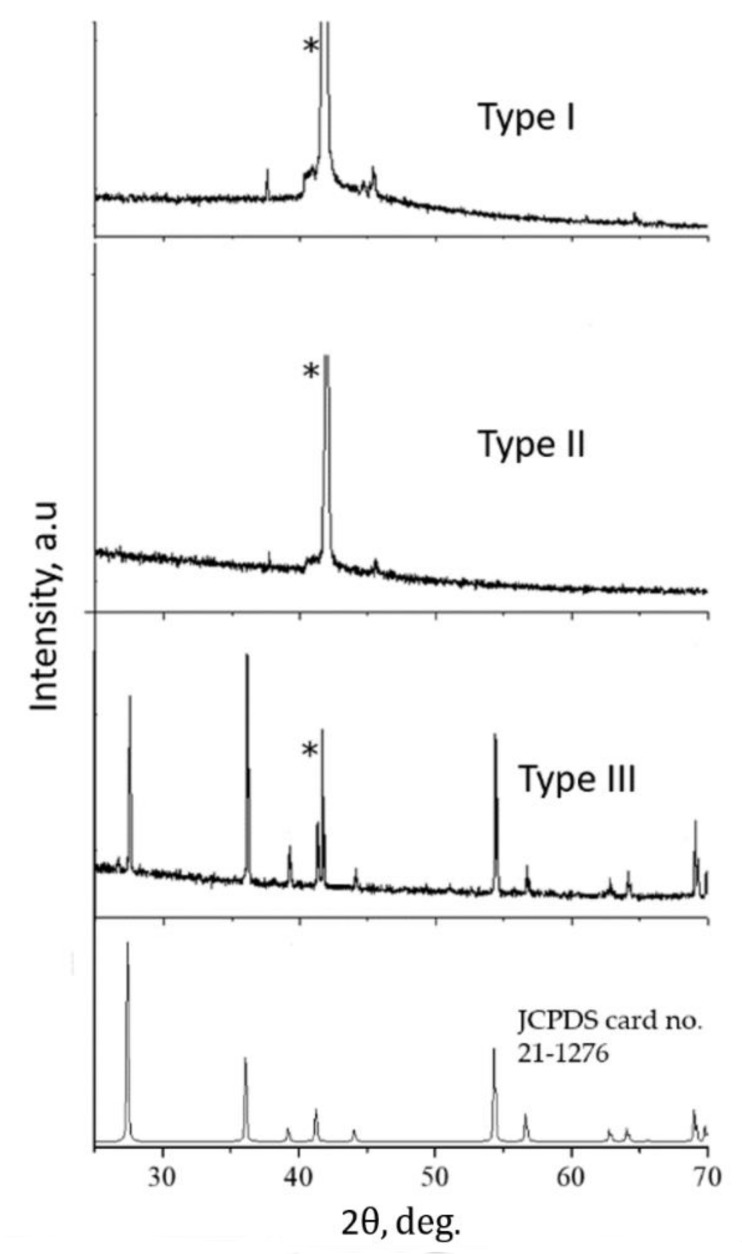
X-ray diffraction pattern of the corresponding samples. * designates reflection of the sapphire substrate.

According to the data obtained, the content of nitrogen in a film of type I is two times lower than in films of types II, III. The nitrogen content in type II and III films differed within the boundaries of measurement error. The XRD patterns of samples processed in all modes are shown in Figure 2. It can be seen that the film samples (types I, II) are X-ray amorphous deposits. In contrast, a polycrystalline rutile phase is formed in the sample (type III) (JCPDS card No. 21-1276). Comparison of the cell parameters shows a slight decrease in the cell parameters: for the standard (JCPDS card No. 21-1276) d_011_ = 0.2487 nm; d_031_ = 0.1360 nm, and for a sample of type III d_011_ = 0.2484 nm; d_031_ = 0.1359 nm. An independent calculation of the lattice deformation gives a value of 0.1%. The data obtained testify to the compression of the rutile lattice. The average crystallite size, according to the Debye–Scherrer formula, is about 119 nm.

**Figure 3:**
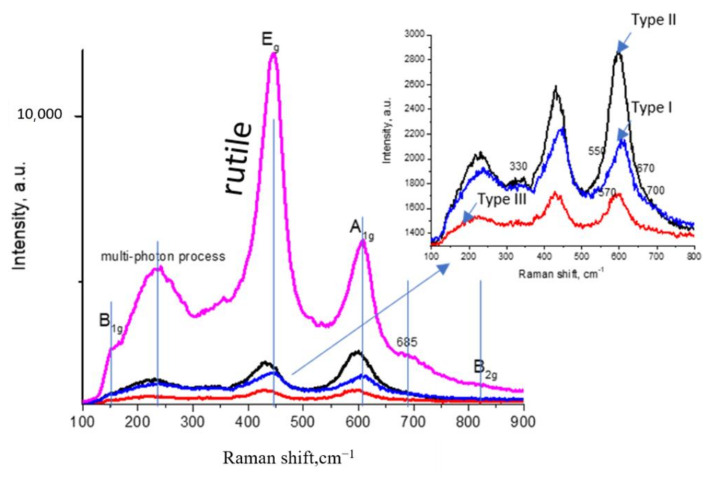
Raman spectrum of the samples of types I, II, and III.

The samples are compared by analyzing Raman spectroscopy data. A rutile film on sapphire exhibits characteristic peaks at 148, 441, 605, 826 cm^−1^ that correspond to the symmetries of B_1g_, E_g_, A_1g_, B_2g_ [29], as well as a peak at 233 cm^−1^ aroused from the multiple phonon scattering processes. The peak at 685 cm^−1^, which accompanies the rutile phase [30], should also be noted. Deconvolution shows the presence of additional peaks at 570, 670 cm^−1^, which correspond to nonstoichiometric TiO_2−x_N_x_ [31], as well as broadened bands at 330, 550, and 700 cm^−1^, which are characteristic of TiN [32,33]. The incorporation of nitrogen atoms can occur both by the type of substitution and that of insertion. When processed in mode III, with the highest mass average plasma temperature and the maximum concentration of active ionized nitrogen atoms, the changes in the lattice parameters (Figure 2) of rutile are negligible. Taking this into account, it can be assumed that the incorporation of nitrogen atoms into the TiO_2_ lattice occurs according to the type of insertion, forming bonds with oxygen (Ti–O–N) [34], as well as the formation of TiN inclusions. The low activity of nitrogen is confirmed by the coincidence of the Raman frequencies of the peaks (Figure 3) of the rutile on the sapphire test film and the type I sample. At the same time, an increase in plasma temperature leads to an increase in the nitrogen content (Table 1) in type II and III samples and a violation of the mixing of the main peaks (Figure 3). Nitrogen-containing phases do not appear on the XRD curves; therefore, the inclusions of titanium nitrides and oxynitrides are mainly contained in the surface layers of the samples.

**Figure 4:**
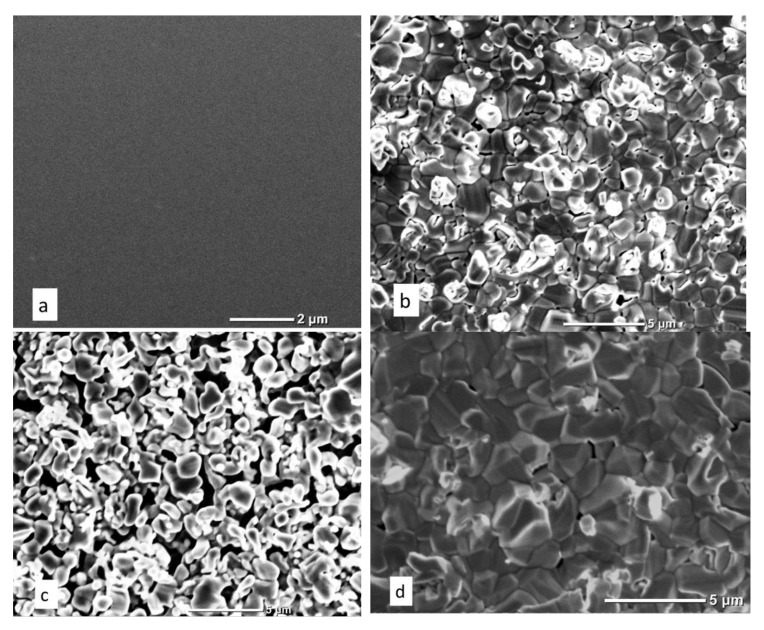
SEM images of sample surface: titanium film on sapphire (**a**); type I (**b**); type II (**c**); type III (**d**).

The XRD results are confirmed by the SEM data (Figure 4). The type III sample contains faceted crystallites up to several micrometers in size. The surface of samples of types I, II contains rounded microstructures without signs of faceting, although rare crystallites up to a micrometer in size are observed.

**Figure 5:**
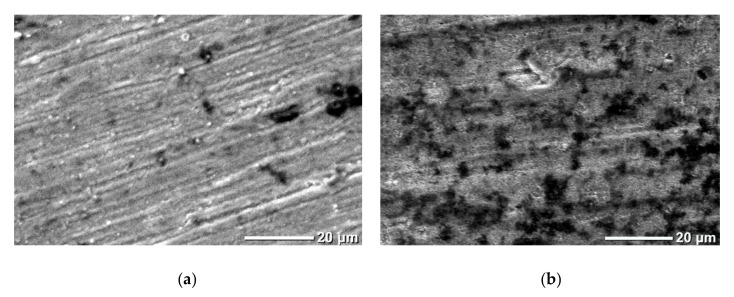
SEM images of the titanium surface: initial (**a**) and after plasma treatment (**b**).

For comparison, polished titanium plates are used in the work. The titanium plate is processed in modes I and III. During treatment in mode III, the titanium surface rapidly oxidizes and, due to high stresses, peels off and disintegrates. However, during processing in mode I, the surface of the plate becomes covered with an oxide layer with good adhesion to the bulk of the sample. The comparison of titanium plates before and after processing (Figure 5) demonstrates a change in surface topography: traces of treatment are absent, etching craters are observed, and roughness increases. According to the EDX data, the nitrogen content in the samples is within the margin of error, while the oxygen concentration is 24 at.%, and titanium 76 at.% (scan region 150 µm × 200 µm). No peculiarities of the composition in the area of dark craters and background are found.

### 3.3. Study of Hardness, Moisture-Resistance, and Raman-Enhancement Properties

#### 3.3.1. Microhardness

**Figure 6:**
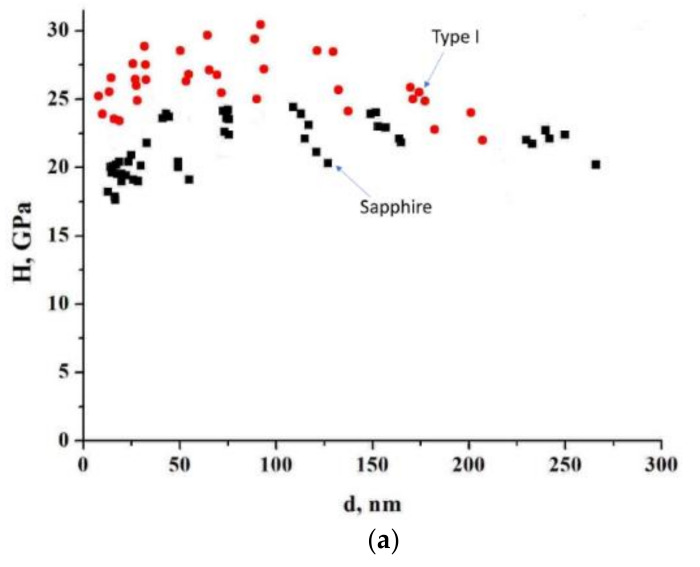

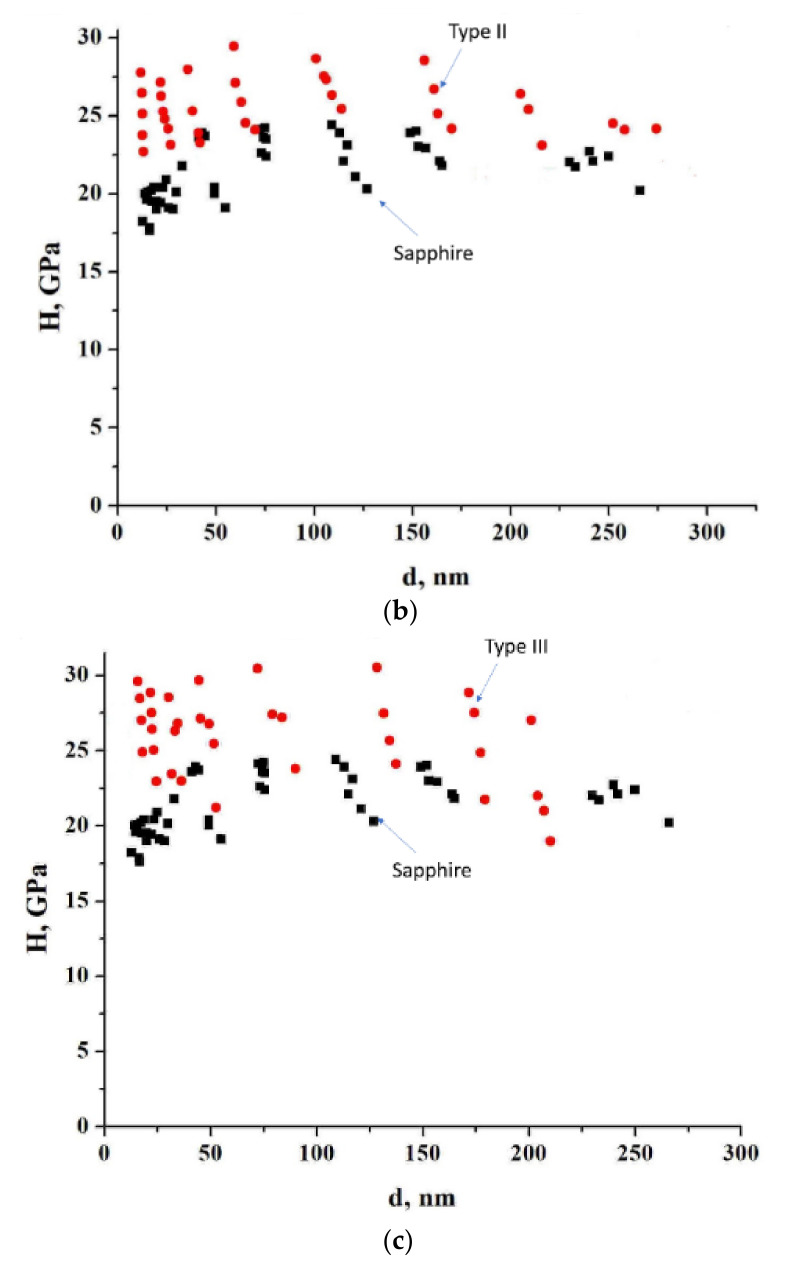
Comparison of the dependence of microhardness on the depth of the indentation of the surface of sapphire and samples of types I (**a**), II (**b**), and III (**c**).

The Vickers hardness of the surface of the sapphire substrate is investigated initially. Vickers hardness (HV) [35] can be calculated according to (1):

HV = 1.8544 *p*/d^2^ (1)

where *p* is the load (in kg) and d average length of two diagonals of the indent (in mm). The microhardness of a sapphire substrate is strongly affected by surface effects (Figure 6) and it is necessary to estimate it by averaging indents in two sectors: those with an indentation depth of up to 50 nm and those with a depth of more than 50 nm. The bulk hardness of the sapphire is determined by the sectors at indentation depths of more than 50 nm and with a value of about 22 GPa, as corresponds to the data obtained [36]. At indentation depths of less than 50 nm, a significant effect is rendered by a disturbed near-surface layer, which is formed as a result of the chemical–mechanical treatment of the sapphire substrate surface. The hardness (nanohardness) of this near-surface layer decreases to 20 GPa.

The average value of the microhardness of the samples treated in different modes is shown in Table 2.

An increase in microhardness can be observed when moving from processing mode I to III. The high value of microhardness for titanium films on sapphire processed in nitrogen plasma should be noted here. Generally speaking, microstructure, porosity, grain boundary surface, stresses, inclusions, etc. can have a decisive influence on microhardness. In our case, the plasma treatment of samples proceeds in an open atmosphere. During plasma treatment, a part of the surrounding atmosphere adjacent to the plasma core is heated. Heating leads to the formation of active oxygen flows to the central axis of the plasma. Under such conditions, oxidation processes are ahead of nitridization processes and this explains the formation of a predominantly titanium oxide phase. An analysis of the literature does not provide a clear conception of the processes of titanium oxidation in the air and their kinetics; however, there are a number of consistencies observable in many experiments. When titanium is oxidized in air in the temperature range of 250–700 °C, the oxidation rate obeys a parabolic law, while in the temperature range of 800–1000 °C, both a linear and parabolic increase in the oxidation rate can be observed, after which it again returns to parabolic [37]. The presence of a porous structure of the film is usually associated with the linear stage, when the diffusion of active oxidant atoms is not limited to a solid/continuous film. As for the parabolic stage, there are several assumptions about limiting processes: diffusion of oxygen ions, or diffusion of metal ions through titanium oxide. The possible presence of a liquid phase during plasma treatment is indicated by rounded particles and pores (Figure 4b,c). Titanium oxidation processes are limited by oxygen diffusion, so they lag behind melting processes. Uneven melting leads to an increase in the local density of the deposit. This may be the reason for the formation of pores and cavities in the film. Particularly high porosity (pore sizes up to several micrometers) is characteristic of a type II sample, indicating the passage of a linear stage of oxidation.

It is likely that nitrogen-containing phases and nitrogen inclusions also have a strong influence. The effect of the nitrogen plasma composition is most pronounced in modes II and III. The coating composition (Table 1) and Raman spectroscopy (Figure 3) confirm the presence of nitrogen-containing phases. The formation of nitrogen-containing phases is associated with the activity of ionized nitrogen atoms, which only increases with increasing temperature. Molecular components, due to the large mass, are less active. A crystalline rutile precipitate is formed only during treatment in mode III (Figure 2 and Figure 4b), which indicates that crystallization conditions have been achieved. The high diffusion activity of the component and temperature contribute to this even with short-term exposure. However, the replacement of oxygen by nitrogen in the TiO_2_ lattice is not observed. The results of the studies indicate the embedded position of nitrogen atoms in the rutile lattice, the segregation of nitrogen at the grain boundaries. Probably, nitrogen-containing phases are initially formed and, with further thermochemical exposure to active oxygen, they are replaced by oxygen and pushed onto the grain boundary. A high content of nitrogen is also observed in the coating formed in mode II, when the temperature and the fraction of ionized nitrogen atoms in the plasma are lower than in mode III. In this case, the coating is an X-ray amorphous precipitate. It can be assumed that a decrease in the concentration of ionized nitrogen atoms and temperature is critical for the formation of a crystalline deposit. Nitrogen is probably released to form TiO_2−x_N_x,_ TiN precipitates, as well as accumulations at grain boundaries. The accumulation of nitrogen at the grain boundaries leads to the restraint of boundary migration and intergranular slip. Similarly, the mobility of dislocations decreases.

Thus, the increase in the microhardness of the films can be associated with the minimum number of dislocation sources in the grains and the predominant effect of the grain-boundary deformation mechanism through the determining role of grain boundaries. The inclusions of the nitrogen-containing phases are mainly concentrated at the grain boundaries, which themselves have a high microhardness [38]. An additional factor may be internal compressive stresses associated with a feature of plasma treatment: a high velocity of the oxidation front from the surface to the depth of the coating.

**Figure 7:**
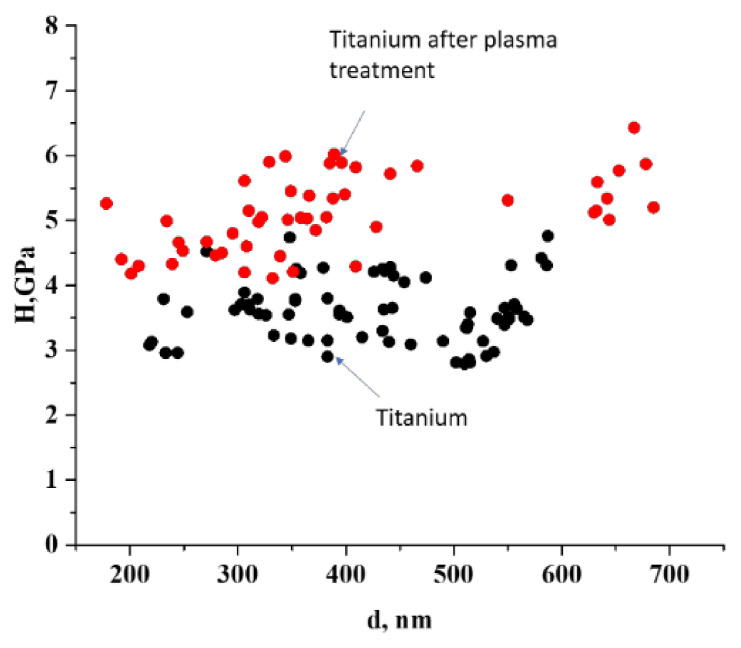
Comparison of the dependence of microhardness on the depth of the titanium surface imprint: initial and after plasma treatment.

As for samples of pure and treated titanium (Figure 5 and Figure 7), the absence of a loose structure characteristic of film samples significantly reduces the diffusion activity of oxygen and nitrogen into the depth of the sample. Processing occurs at the minimum mass-average plasma temperature, which explains the absence of nitrogen according to the EDX data. However, partial incorporation of oxygen occurs and, due to this, a near-surface phase of titanium oxide with a low grain size is formed. The presence of such a coating promotes an increase in microhardness up to 5.5 GPa.

#### 3.3.2. Moisture-Resistant Properties

**Figure 8:**
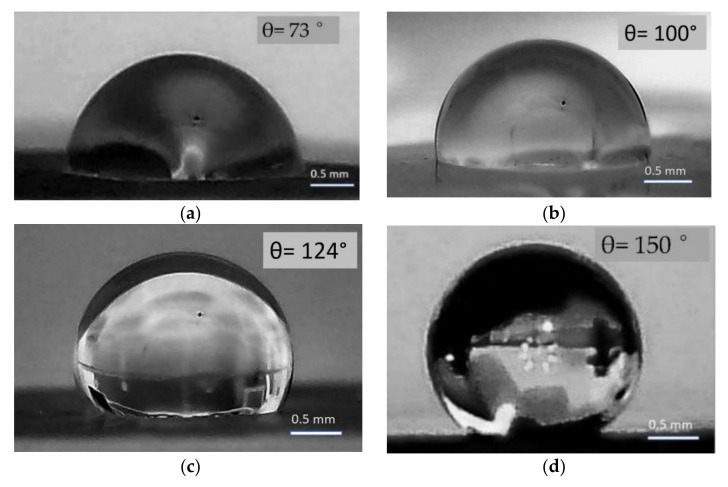
Optical images of the shape of a water drop on the surface of the samples: type I (**a**), II (**b**), III (**c**,**d**).

The study of moisture-resistant properties reveals an interesting result: with an increase in the mass-average plasma temperature, the state of the surface transforms from hydrophilic to hydrophobic (Figure 8). The wetting angle θ for samples of type I and II is 73° and 100°, respectively. Different values of the wetting angle are obtained for the type III sample, depending on the surface area: from hydrophobic 124° to super-hydrophobic 150°.

The results obtained indicate significant differences in the processes of oxidation and crystallization of titanium film on sapphire, depending on the processing conditions. It is apparent that during treatment in mode III, all processes proceed predominantly in the solid phase. The high temperature (7–10 kK) contributes to the rapid heating of the surrounding atmosphere and, as a consequence, to the high activity of oxygen, as well as ionized plasma atoms. It is not possible to implement the processes of solid-phase epitaxy with the formation of an epitaxial rutile film on sapphire, since a longer duration of plasma treatment leads to the destruction of the substrate. However, the processing conditions are sufficient to form a polycrystalline rutile phase. Otherwise, the processing of samples in modes I and II leads to the formation of an amorphous structure of titanium oxide with a porous surface and rounded formations. The presence of such topographic irregularities is associated with an increase in the root mean square roughness R_q_ of films of types I and II, 136.4 nm and 148.3 nm, respectively. For a type III crystalline sample, the R_q_ = 71.4 nm. Thus, there is no regular correlation between the surface roughness value for samples of types II and III and the wetting angle: for hydrophobic samples in which the Cassie state is realized, a decrease in roughness should lead to a decrease in the wetting angle. However, the increase in the hydrophobic component of type I and II samples is probably related to the surface roughness and porosity. The difference in contact wetting angles for a type III crystalline sample is due to the strong exposure when processed in mode III, which is the most difficult to control: the microrelief is uniform, but the macrorelief includes unevenly distributed craters.

It is possible that the moisture-resistant properties are significantly affected by the nitrogen-containing phases and the crystal structure of the surface. It should be noted that there is a small amount of experimental data investigating the effect of nitrogen impurities and its phase (incorporation, substitution) state on the hydrophobic properties of the surface. The calculated data [39] show that when TiO_2_ clusters are doped with nitrogen, the positive energy of Gibb’s adsorption increases, which can contribute to an increase in hydrophobicity. In addition, the close-to-super-hydrophobic nature of the coating obtained in mode III can be associated with its monophase structure [16], as well as the possible formation on the surface of titanium dioxide of an ordered molecular monolayer of carboxylic acids with pronounced hydrophobic properties [40].

#### 3.3.3. Raman Active Properties

**Figure 9:**
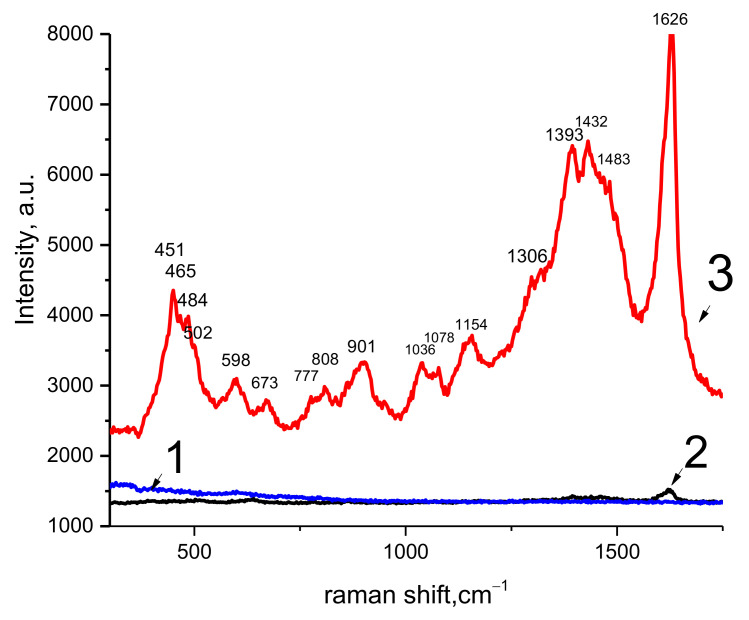
Raman spectra of the MB film deposited on I (1), II (2), III (3) samples.

Raman-enhancement studies of the properties of the samples are presented in Figure 8. When probing the MB film, slightly noticeable bands and peaks are observed on the surface of samples of types I and II. Moreover, in a sample of type I, bands of various titanium oxides appear, located mainly in the short-frequency region of the spectrum, and for a sample of type II, peaks in the far region of 1393, 1464, and 1621 cm^−1^, corresponding to the MB film [41]. The most intense and contrasting peaks of the MB film are observed for the type III sample (Figure 9, curve 2). It should be noted that the Raman peaks corresponding to the MB film appeared only in samples of types II and III, in which the content of nitrogen-containing phases is maximum. The picture of the enhanced Raman spectrum is observed only for a type III sample, which differed in crystallinity. Thus, the features of the Raman spectra of the MB film on type II and III samples can be associated with surface-enhanced resonant Raman scattering due to the effective excitation of surface plasmon polaritons on TiN particles in the TiO_2_ matrix. The multiple increases in the intensity of the Raman spectrum of MB in the case of using a polycrystalline rutile matrix is associated with an increase in the dephasing time of localized surface plasmons. The dephasing time determines [42] the quality of the resonance and the achievable amplification of the electromagnetic field near TiN inclusions. The lattice defects of the TiO_2_ matrix reduce the dephasing time of plasmons and degrade the quality of the resonance, which manifests itself in the case of a type II sample. For a type I sample with a minimum TiN content and an amorphous base, the dephasing time is minimized, so that no resonance phenomena are observed. The relative gain calculated from the intensity of the 1626 cm^−1^ peak for type II and III samples is 40.

## 4. Conclusions

The paper shows the prospects of treatment with a quasi-equilibrium low-temperature nitrogen plasma in an open atmosphere for the formation of super-hard, super-hydrophobic TiN/TiO_2_ composite coatings with pronounced Raman-enhancing properties. During the plasma treatment of titanium films on sapphire with a mass average temperature of 4–6 kK, an amorphous hydrophilic titanium oxide film with a low nitrogen content is formed. The nitrogen content in titanium oxide films increases with increasing treatment temperature up to 6–7 kK. In this case, an X-ray amorphous hydrophobic film is formed. With a further increase in temperature to 7–10 kK, a TiN/TiO_2_ composite structure based on polycrystalline rutile is formed with increased hydrophobicity and pronounced Raman-enhancing properties due to the effective excitation of surface plasmon polaritons. The presence of the crystalline phase increases the dephasing time, which determines the quality of the resonance and the achievable amplification of the electromagnetic field near the TiN inclusions. All treated titanium films on sapphire have a super-hardness above 25 GPa (Vickers hardness test) due to high grain size, the presence of nitrogen-containing inclusions concentrated along grain boundaries, and compressive stresses. When processing clean titanium plates in plasma with a mass-average temperature of 4–6 kK, the surface is partially oxidized and the hardness increases to 5.5 GPa.

## Figures and Tables

**Table 1:** Ratio of elements according to EDX data.

	Type I	Type II	Type III
N/Ti	0.22	0.51	0.52

**Table 2:** Average value of the microhardness of the samples treated in different modes.

Sample	Type I	Type II	Type III
Hardness, GPa	23.9	26.0	27.2
Sample	pristine titanium		treated titanium
Hardness, GPa	3.5		5.5
